# The Role of IGF‐1 and IL‐10 in Childhood Stunting: Evidence From Indonesia

**DOI:** 10.1155/tswj/1029295

**Published:** 2026-08-13

**Authors:** Putriatri Krimasusini Senudin, Irwanto, Nur Aisiyah Widjaja, Sulistiawati

**Affiliations:** ^1^ Doctoral Program of Medical Science, Faculty of Medicine, Universitas Airlangga, Surabaya, Indonesia, unair.ac.id; ^2^ Department of Midwifery, Faculty of Health Science, Universitas Katolik Indonesia Santu Paulus Ruteng, Kabupaten Manggarai, Indonesia; ^3^ Department of Child Health, Faculty of Medicine, Universitas Airlangga, Dr. Soetomo General Academic Hospital, Surabaya, Indonesia, unair.ac.id; ^4^ Departement of Public Health and Preventive Medicine, Faculty of Medicine, Universitas Airlangga, Surabaya, Indonesia, unair.ac.id

**Keywords:** child growth, early childhood, IGF-1, IL-10, nutritional status, stunting

## Abstract

Stunting remains a pressing public health problem in low‐ and middle‐income countries. The specific biochemical roles of growth‐ and immune‐related biomarkers with stunting are less understood. This study is aimed at investigating the contribution of insulin‐like growth factor 1 (IGF‐1) and interleukin‐10 (IL‐10) to stunting among Indonesian children. A case‐control design was employed involving 80 children aged 24–60 months in Manggarai, Indonesia (40 stunted and 40 nonstunted), matched by age, sex, and residence. Data were collected through anthropometric measurements, household surveys, structured interviews, and biochemical analysis of serum IGF‐1 and IL‐10 using ELISA. Findings revealed that stunted children had significantly lower IGF‐1 levels (11.963 ± 8.658 vs. 18.000 ± 15.238, *p* < 0.05) and higher IL‐10 levels (317.841 ± 214.934 vs. 543.605 ± 416.837, *p* < 0.05) compared with controls. The study concludes that effective stunting prevention requires integrated interventions addressing both nutritional adequacy and infection control in early childhood.

## 1. Introduction

Stunting is a major global public health issue affecting millions of children worldwide, particularly in low‐ and middle‐income countries such as Indonesia. It reflects chronic undernutrition and caused long‐term deficits in cognitive development, physical growth, and future productivity. In Indonesia, stunting is prevalent, with substantial variations across different regions and socioeconomic groups.

In 2013, approximately 8.9 million (37.2%) of all Indonesian children under five were stunted in 2013 [1]. Despite efforts to reduce these numbers, stunting persists, with a 23% prevalence in Malang Regency in 2022 [2]. Stunting leads to high mortality rates, impaired cognition, poor academic performance, and reduced productivity in adulthood [1, 3].

Several factors contribute to the high prevalence of stunting in Indonesia, particularly in regions such as Manggarai. Socioeconomic determinants include maternal education, family income, and employment status. Children of uneducated mothers face a higher risk of moderate and severe stunting than those of tertiary‐educated mothers [4, 5]. Additionally, low family income and unemployment among mothers are associated with higher stunting rates [2, 6].

Health‐related factors such as low birth weight, inadequate antenatal care, and a history of infectious diseases strongly affect stunting. Children with low birth weight have higher odds of severe and moderate stunting [2, 4, 7]. Furthermore, inadequate antenatal care and maternal health during pregnancy, including anemia and lack of immunization, increase stunting risk [2, 4, 8].

Environmental factors such as poor sanitation and lack of clean water exacerbate stunting. Inadequate water, sanitation, and hygiene account for 56% of malnutrition cases, highlighting the need for safe drinking water and sanitation investments [1, 9]. Food insecurity and limited access to nutritious food further increase stunting risk [10, 11].

Stunting, a condition characterized by impaired child growth and development, is influenced by a complex interplay of biological, nutritional, and environmental factors. Recent studies emphasize the role of biological factors, including growth‐regulating hormones and immune responses in contributing to stunting, alongside traditional factors such as nutritional intake and environmental conditions [12–14]. For example, infections, even asymptomatic, trigger systemic inflammation that reduces growth‐promoting hormone levels such as insulin‐like growth factor 1 (IGF‐1), hindering growth [15]. Addressing stunting requires a holistic approach that includes biological and traditional risk factors.

IGF‐1 is a crucial hormone essential for linear growth and development. IGF‐1 regulates various cellular processes including proliferation, differentiation, and apoptosis [16–18]. Primarily produced in the liver, IGF‐1 acts on bones, muscles, and the nervous system [19, 20]. The hormone′s bioavailability is regulated by IGF‐binding proteins (IGFBPs), which modulate its interaction with the IGF‐1 receptor [21]. Disruptions in the IGF‐1 signaling pathway can lead to growth impairments and are linked to conditions such as intrauterine growth restriction and various metabolic diseases [22, 23]. Therefore, IGF‐1 is essential for normal growth and a potential therapeutic target for growth‐related disorders.

Moreover, interleukin‐10 (IL‐10) is an anti‐inflammatory cytokine essential for modulating immune responses. It primarily suppresses inflammation by inhibiting the production of proinflammatory cytokines and other inflammatory mediators [24–26]. IL‐10 achieves this via the Jak1/Tyk2 and STAT3 signaling pathways, maintaining cellular homeostasis and preventing tissue damage during immune responses [24]. Notably, IL‐10 also has immunostimulatory properties under certain conditions, enhancing immune responses against infections [27, 28]. Its dual suppressive and stimulatory roles make IL‐10 crucial in managing hyperinflammatory conditions such as autoimmune diseases and chronic infections [29, 30]. Understanding the regulatory mechanisms of IL‐10 may inform novel therapeutic strategies aimed at modulating immune responses more effectively.

Although nutritional and socioeconomic determinants of stunting are well studied, the biochemical contributions, particularly the roles of IGF‐1 and IL‐10, remain underexplored in Indonesian children. IGF‐1 is crucial for growth and development, with low levels associated with stunting in children. For example, a study conducted in Surabaya, Indonesia, showed stunted children had significantly lower IGF‐1 levels than nonstunted children, suggesting a protective role [31]. Similarly, another study found high blood lead levels, which disrupt IGF‐1, increase stunting risk in children living in agricultural areas [32]. These findings highlight IGF‐1′s importance in growth and its potential vulnerability to environmental factors.

The role of IL‐10 in stunting among Indonesian children remains unclear. Although IL‐10 has anti‐inflammatory properties, its specific impact on stunting is poorly documented in Indonesian children. A study on Bangladeshi children indicated that IL‐10 levels significantly increased among stunted children after intervention, suggesting a potential role in the inflammatory response associated with stunting [33]. Despite this, a notable gap exists in research specifically addressing the biochemical pathways involving IL‐10 in Indonesian children. Therefore, future studies should elucidate the roles of IGF‐1 and IL‐10 to provide a comprehensive understanding of the biochemical factors contributing to stunting in this population. This study addresses this gap by investigating the contribution of IGF‐1 and IL‐10 levels to stunting among children aged 24–60 months in Puskesmas Kota, Manggarai, Indonesia, using a case‐control design.

## 2. Materials and Methods

### 2.1. Study Design and Participants

This study employed a case‐control design to investigate the role of serum IGF‐1 and IL‐10 levels in stunting incidence among children aged 24–60 months. A case‐control approach is suitable for assessing exposure‐outcome associations, particularly when outcomes are uncommon or when ethical or logistical considerations prevent prospective cohort designs [34]. Its retrospective nature enables efficient examination of multiple predictors within a defined population [35, 36].

This study was conducted in the working area of Puskesmas Kota, located in Manggarai Regency, East Nusa Tenggara, Indonesia, which serves 11 urban villages: Laci, Carep, Compang Carep, Bangka Nekang, Karot, Mbaumuku, Pitak, Pocomal, Satar Tacik, Tadong, and Watu. The study was implemented over 4 months (September to December 2024). Its primary objective was to examine and compare serum IGF‐1 and IL‐10 levels between stunted and nonstunted children.

The study population included all children aged 24–60 months in the designated Puskesmas Kota area. Eighty children, boys and girls, were recruited via random sampling. The sample included 40 stunted (defined by a height‐for‐age *Z*‐score [HAZ/LAZ] < −2 standard deviations [SDs]) and 40 nonstunted children (HAZ/LAZ ≥ −2 SD). Participants in the control group were matched to cases based on residential location, age, and sex, following a 1:1 case‐control ratio.

Data were collected by conducting structured interviews and household surveys with parents for the case and control groups. Information on infectious disease history was gathered using a standardized questionnaire developed by the Indonesian Ministry of Health (2018). Nutritional intake was assessed using a 24‐h dietary recall instrument adapted from the 2018 Indonesian National Food and Nutrition Workshop. Dietary data were subsequently processed and analyzed using Nutrisurvey 2007.

### 2.2. Anthropometric Measurements

Body weight was measured using either a SECA 334 infant scale or a SECA 813 standing scale (SECA GmbH, Hamburg, Germany) and recorded in grams. Height or recumbent length was measured with a SECA 416 infantometer or SECA 213 stadiometer and recorded in centimeters. Anthropometric indices, including HAZ/LAZ, were calculated using the World Health Organization (WHO) child growth standards. Stunting was classified as a HAZ/LAZ score below −2 SD.

### 2.3. Biochemical Analysis

Venous blood samples (5 mL) were collected from each participant in ethylenediaminetetraacetic acid tubes by trained local laboratory personnel. Samples were centrifuged at 4000 rpm for 10 min, and serum was promptly separated and stored at −20°C until further analysis. IGF‐1 and IL‐10 concentrations were measured using enzyme‐linked immunosorbent assay (ELISA) kits following the manufacturer′s protocols (Bioassay Technology Laboratory, Diagnostic Product Corp, BT LAB, China). Specifically, the IGF‐1 ELISA (E0103Hu) and IL‐10 ELISA (E0102Hu) kits were used. Absorbance readings were recorded using an ELISA microplate reader (Model 17539), and biomarker concentrations were determined against provided standards.

### 2.4. Statistical Analyses

Statistical analyses were conducted using IBM SPSS Statistics Version 20.0. Descriptive statistics were reported as the mean ± SD for continuous variables or as frequencies and percentages for categorical variables. Group comparisons between stunted and nonstunted children were performed using the chi‐square test for categorical variables (e.g., sex, exclusive breastfeeding, immunization status, nutritional intake, and infectious disease history) and the Mann–Whitney *U* test for continuous variables (e.g., age, IGF‐1, and IL‐10 levels). Statistical significance was set at *p* < 0.05.

### 2.5. Ethical Considerations

This study was approved by the Health Research Ethics Committee of the Faculty of Medicine, Universitas Airlangga, Surabaya (approval number 203/EC/KEPK/FKUA/2024).

## 3. Results

This study included 80 children between 24 and 60 months who met the inclusion criteria. The mean age of children in the stunted group was 38.52 ± 8.99 months, and the mean age of children in the normal group was 37.35 ± 10.63 months. No statistically significant difference in age was observed between the two groups (*p* = 0.473). Similarly, sex distribution was comparable across both groups (*p* = 0.451). The stunted group included 21 males (52.5%) and 19 females (47.5%), whereas the normal group consisted of 17 males (42.5%) and 23 females (57.5%).

The stunted group demonstrated lower average weight and height than their normal counterparts, with mean values of 10.56 ± 1.92 kg and 83.69 ± 6.74 cm, respectively, versus 14.18 ± 2.11 kg and 95.47 ± 6.20 cm, respectively, in the normal group.

Potential factors influencing IGF‐1 and IL‐10 levels were also examined, including history of exclusive breastfeeding, immunization status, dietary intake of carbohydrates and proteins, and infectious disease history. The results indicated no significant differences between the two groups for exclusive breastfeeding (*p* = 0.815), immunization history (*p* = 0.474), and protein intake (*p* = 0.314). However, among the infectious diseases considered, only acute respiratory infections showed a statistically significant difference between the groups (*p* = 0.000). In contrast, other illnesses such as pneumonia (*p* = 0.474), tuberculosis (*p* = 0.314), and diarrhea (*p* = 0.348) did not exhibit significant differences.

A significant disparity was observed in IL‐10 levels, with lower averages detected in the stunted group than the normal group (317.841 ± 214.934 vs. 543.605 ± 416.837; *p* = 0.006). Similarly, IGF‐1 levels in stunted children were significantly reduced compared with those in the normal group (11.963 ± 8.658 vs. 18.000 ± 15.238; *p* = 0.030) (see Table 1).

**Table 1:** Respondent characteristics.

Characteristics	Stunting group (*n* = 40)	Normal group (*n* = 40)	*p*
Age (months)	38.52 ± 8.99	37.35 ± 10.63	0.473
Gender			
Man	21 (52.5%)	17 (42.5%)	0.451
Woman	19 (47.5%)	23 (57.5%)	
Body weight (kg)	10.56 ± 1.92	14.18 ± 2.11	0.000^b^ ^∗^
Height (cm)	83.69 ± 6.74	95.47 ± 6.20	0.000^b^ ^∗^
Exclusive breastfeeding			
Yes	25 (62.5%)	27 (67.5%)	0.815
No	15 (37.5%)	13 (32.5%)	
Immunization			
Complete	38 (95.0%)	40 (100.0%)	0.474
Incomplete	2 (5.0%)	0	
Protein intake			
Adequate	39 (97.5%)	40 (100%)	0.314
Inadequate	1 (2.5%)	0	
Carbohydrate intake			
Adequate	22 (55.0%)	38 (95.0%)	0.000^a^ ^∗^
Inadequate	18 (45.0%)	2 (5.0%)	
History of infectious diseases			
Upper respiratory tract infection			
Yes	30 (75.0%)	10 (25.0%)	0.000^a^ ^∗^
No	10 (25.0%)	30 (75.0%)	
Pneumonia			
Yes	2 (5.0%)	0	0.474
No	38 (95.0%)	40 (100.0%)	
Tuberculosis			
Yes			
No			
Diarrhea			
Yes	8 (20.0%)	4 (10.0%)	0.348
No	32 (80.0%)	36 (90.0%)	
IL‐10 (pg/mL)	317,841 ± 214,934	543,605 ± 416,837	*0.006* ^∗^ *^b^*
IGF‐1 (ng/mL)	11,963 ± 8658	18,000 ± 15,238	*0.030* ^∗^ *^b^*

*Note:* mean ± SD. The italic values indicate statistically significant differences (*p* < 0.05).

Abbreviation: SD = standard deviation.

^a^Chi‐square test.

^b^Whitney *U* test.

^∗^ *p* < 0.05.

Table 2 shows a statistically significant association between IGF‐1 and IL‐10 levels and the likelihood of stunting among children aged 24–60 months. Logistic regression analysis revealed that IGF‐1 levels were significantly associated with stunting (*p* = 0.032), with an odds ratio (OR) of 1.049 and a 95% confidence interval (CI) in the range of 1.004–1.069. This suggested that for every one‐unit (nanograms per milliliter) increase in IGF‐1, the odds of a child being stunted increase by approximately 4.9%, assuming all other variables remain constant.

**Table 2:** Logistic regression of IGF‐1 and IL‐10 with stunting.

Variables	*p* value	OR	95% CI	
			Lower	Upper
IGF‐1 (ng/mL)	0.032	1.049	1.004	1.069
IL‐10 (ng/mL)	0.010	1.002	1.001	1.004

Abbreviations: CI = confidence interval; OR = odds ratio.

Similarly, IL‐10 levels also demonstrated a statistically significant relationship with stunting (*p* = 0.010), with an OR of 1.002 and a 95% CI in the range of 1.001–1.004. Although the OR is modest, it implied that even small increases in IL‐10 concentrations are associated with higher odds of stunting. The narrow CIs for both biomarkers emphasized estimate precision.

Collectively, these findings suggested that elevated IGF‐1 and IL‐10 levels are positively associated with stunting risk, contrary to common expectations for IGF‐1. This warrants further investigation into the complex biological and environmental mechanisms underlying these associations.

## 4. Discussion

Stunting is a major global health issue, affecting approximately 149 million children under five, particularly prevalent in low‐ and middle‐income countries [37, 38]. The condition results from chronic malnutrition and frequent infections during the critical first 1000 days of life, leading to impaired physical growth, cognitive deficits, and increased susceptibility to chronic diseases later in life [39, 40]. Despite various maternal and child health strategies aimed at its reduction, stunting prevalence remains high with notable socioeconomic disparities across different groups [41, 42]. The long‐term impacts of stunting include reduced cognition, motor development, economic productivity, and increased morbidity and mortality rates, reinforcing poverty and socioeconomic decline [39, 43]. Effective reduction requires comprehensive, multisectoral strategies targeting maternal and child nutrition, infection prevention, and socioeconomic support [40, 44].

Thus, this study highlights the roles of IGF‐1 and IL‐10 in stunting. Our findings indicated that hormonal and immunological factors contribute to growth impairments in children, highlighting stunting′s multifactorial nature.

Consistent with prior research, this study showed significantly lower IGF‐1 levels in stunted children than in their nonstunted peers (11.963 ± 8.658 vs. 18.000 ± 15.238; *p* = 0.030). IGF‐1 is a critical growth factor, and its deficiency is linked to impaired growth and development in children. Stunted children often have considerably IGF‐1 lower levels than their nonstunted peers [31, 45]. This hormone promotes linear growth, and its deficiency may result from various factors, including poor nutrition and chronic infections [31, 45]. For example, systemic inflammation in early infancy can suppress the growth hormone‐IGF axis, leading to stunting [45]. Additionally, interventions aimed at increasing IGF‐1 levels, such as vitamin D supplementation, improve growth outcomes in children with idiopathic short stature [46]. Therefore, maintaining adequate IGF‐1 levels is crucial for preventing stunting, particularly in children aged 24–60 months who are at a critical stage of growth and development.

Moreover, high IL‐10, an anti‐inflammatory cytokine, has also been implicated in increased stunting, particularly in children with upper respiratory tract infections (URTIs). IL‐10 regulates immune responses and limits inflammation [47, 48]. However, elevated IL‐10 levels during infections can increase susceptibility to infections and impair growth [47, 48]. For example, children with recurrent URTIs and high IL‐10 levels may experience more severe infections, negatively impacting growth [47, 48]. Moreover, high IL‐10 levels during respiratory infections are associated with conditions such as bronchiolitis, further hindering growth in children [47, 49]. Thus, although IL‐10 controls inflammation, its elevation in frequent infections may contribute to stunting.

The interplay among nutritional intake, infections, and growth is evident in the roles of IGF‐1 and IL‐10. Poor nutritional intake, particularly inadequate carbohydrate consumption, can lead to low IGF‐1 levels, increasing stunting risk [31, 45]. Concurrently, frequent infections, such as URTIs, elevate IL‐10 levels, exacerbating growth impairment risk [47, 48]. This dual burden of malnutrition and infections creates a vicious cycle that hinders optimal growth and development in children. Interventions targeting both nutritional support and infection control are crucial to breaking this cycle and promoting healthy growth.

Low IGF‐1 levels have been consistently associated with stunting in children. Several studies confirm IGF‐1′s crucial role in growth and development. For example, children with transfusion‐dependent thalassemia and low IGF‐1 levels are more likely to be stunted compared with those with higher IGF‐1 levels [50]. Similarly, idiopathic short stature in children has been linked to low IGF‐1 levels, which improve after Vitamin D supplementation [46]. This suggests that nutritional interventions targeting IGF‐1 levels may help mitigate stunting. Furthermore, stunted children have lower IGF‐1 levels than their nonstunted counterparts, reinforcing the importance of IGF‐1 in linear growth [31, 33, 51–56]. IGF‐1 correlates with growth parameters such as height and weight, highlighting its potential as a biomarker for stunting [53, 55, 56]. Therefore, addressing IGF‐1 deficiency via dietary or medical interventions could be a strategic approach to reduce stunting prevalence.

High IL‐10 levels have been linked to stunting, particularly in children with URTIs. IL‐10 modulates immune responses and is elevated in stunted children [33, 57–63]. For example, children with infection‐related growth failure show changes in IL‐10 levels after nutritional interventions [62], suggesting its role in immune dysregulation and stunting. Additionally, systemic inflammation marked by elevated IL‐10 is associated with stunting [33, 61, 63]. High IL‐10 levels in stunted children with infections indicate a complex interplay between immune function and growth impairment. Moreover, the relationship of IL‐10 with other inflammatory markers such as IL‐6 and TNF‐*α* highlights the importance of immune responses in stunting [33, 57–63]. Therefore, interventions targeting IL‐10 modulation and immune function may help address infection‐associated stunting.

Therefore, addressing both IGF‐1 deficiency and immune dysregulation, particularly elevated IL‐10 levels, may be critical for reducing stunting prevalence in children. Nutritional interventions to increase IGF‐1 levels and strategies to modulate immune responses may offer promising avenues for improving growth outcomes in affected populations. The association of low IGF‐1 and high IL‐10 with increased stunting in children has considerable public health implications. Low IGF‐1 levels, linked to inadequate carbohydrate intake and primarily affecting children aged 24–60 months, suggest the need for nutritional interventions to improve carbohydrate intake and overall diet quality in stunting prevention [31, 45, 64]. Meanwhile, high IL‐10 levels, associated with URTIs, indicate that reducing infection incidence via improved sanitation, vaccination, and healthcare access could mitigate stunting [65, 66]. Therefore, public health strategies should include nutritional programs ensuring adequate carbohydrate intake and balanced nutrition for children, especially those aged 24–60 months. Strategies should also enhance healthcare access to prevent and treat infections and improve sanitation and hygiene practices to reduce infection‐related inflammation. Education and awareness campaigns should target caregivers and communities on the importance of nutrition and infection prevention. These integrated strategies, if effectively implemented, could substantially reduce stunting prevalence and improve long‐term health outcomes for children in affected regions.

This study had limitations, including a small sample size that may limit generalizability. Future research should elucidate the relationship among IGF‐1, IL‐10, and stunting. Longitudinal studies are essential to track IGF‐1 and IL‐10 level changes over time and their direct impact on growth patterns in children [31, 33]. Intervention trials should explore the effects of administering IGF‐1 and IL‐10, separately or combined, on stunting and growth outcomes [33, 67]. Additionally, research should investigate the role of other cytokines and growth factors, such as IL‐6, TNF‐*α*, and IGFBPs, that interact with the IGF system and influence growth [68–70]. Understanding these interactions may elucidate the biological mechanisms underlying stunting and identify potential therapeutic targets.

## 5. Conclusion

This study highlights the roles of hormonal and immunological factors in stunting among children, particularly the association among low IGF‐1, high IL‐10, and poor growth outcomes. Low IGF‐1 levels, linked to inadequate carbohydrate intake in children aged 24–60 months, highlight the need for early nutritional interventions in mitigating stunting during the critical developmental period. Concurrently, high IL‐10 levels, associated with frequent URTIs, reflect immune dysregulation that may further hinder growth, emphasizing the impact of infection‐related inflammation on child development.

These findings affirm the multifactorial nature of stunting and highlight the need for integrated public health strategies addressing both nutritional deficiencies and infection control. Effective prevention requires nutritional programs aimed at improving diet quality and macronutrient adequacy, particularly carbohydrate intake, alongside improved access to healthcare, sanitation, and immunization services. Despite contributing evidence on biological stunting risk factors, the study′s small sample size highlights the need for further longitudinal and interventional research. Future studies should explore broader immunological and endocrine profiles to better understand complex stunting mechanisms and inform more targeted, evidence‐based interventions.

## Author Contributions

Conceptualization: P.K.S. and I. Methodology and data analysis: N.A.W. and S. Writing—preparation of initial draft: P.K.S. Writing—review and validation: I., N.A.W., and S.

## Funding

No funding was received for this manuscript.

## Ethics Statement

This research was approved by the Health Research Ethics Committee of the Faculty of Medicine, Airlangga University, Surabaya, with Number 203/EC/KEPK/FKUA/2024.

## Consent

Written informed consent was obtained from the parents or legal guardians of all pediatric participants prior to enrollment in the study.

## Conflicts of Interest

The authors declare no conflicts of interest.

## Data Availability

All data generated or analyzed during this study are included in this published article. The datasets supporting the conclusions of this research are presented within the tables provided in the manuscript. No additional datasets were used or created, and the authors confirm that the data are sufficient to replicate the findings reported herein.
